# Clinical implication and management of rectal cancer with clinically suspicious lateral pelvic lymph node metastasis: A radiation oncologist’s perspective

**DOI:** 10.3389/fonc.2022.960527

**Published:** 2022-12-07

**Authors:** Gyu Sang Yoo, Hee Chul Park, Jeong Il Yu

**Affiliations:** Department of Radiation Oncology, Samsung Medical Center, Sungkyunkwan University School of Medicine, Seoul, South Korea

**Keywords:** rectal neoplasm, lateral pelvic lymph node, recurrence, lymph node dissection, neoadjuvant treatment, radiotherapy, chemoradiation

## Abstract

Rectal cancer is the eighth most common malignancy worldwide. With the introduction of total mesorectal excision (TME) and neoadjuvant chemoradiation (NCRT), intrapelvic local control has been remarkably improved. However, lateral pelvic recurrence remains problematic, especially in patients with clinically suspicious lateral pelvic lymph node (LPLN). LPLN dissection has been applied for the management of LPLN metastasis, mainly in Japan and other Eastern countries, while the role of NCRT is more emphasized and LPLN dissection is performed in very limited cases in Western countries. However, the optimal management strategy for patients with rectal cancer with suspicious LPLN metastasis has not been determined. Herein, we review the latest studies on the optimal management of LPLN metastasis to suggest the most appropriate treatment policies according to current evidence and discuss future research directions.

## Introduction

Rectal cancer is the eighth most common malignancy and has a worldwide incidence of 3.2% (1). The rate of pelvic recurrence after surgery for rectal cancer was previously reported to be high (approximately 40%) (2). However, after total mesorectal excision (TME) was introduced, the recurrence rates fell dramatically to 6.5% (3, 4). TME enables a sufficient circumferential margin and the removal of the perirectal lymph nodes embedded in the mesorectum (3). Therefore, it can reduce the probability of residual malignant cells compared with pre-TME surgery, which was more prone to leaving malignant tumor cells in the residual mesorectal tissues. In addition to TME, the application of radiation therapy (RT) and chemotherapy as an adjuvant or neoadjuvant treatment has improved intrapelvic control even after the introduction of TME, particularly in patients with locally advanced, low-lying rectal cancer (5–7).

Despite the remarkable improvements in terms of intrapelvic control in the management of rectal cancer with the application of these multimodal treatments, pelvic side wall recurrence remains a significant concern, especially in patients with clinically suspicious lateral pelvic lymph node (LPLN) metastasis or its potential risk factors, such as location below the peritoneal reflection, advanced T and/or N stage, or radiological size of the LPLN (8, 9). Because the area of LPLN is outside the field of TME, some studies from Eastern countries have suggested that LPLN dissection (LPLND) in addition to TME is required to achieve sufficient pelvic control in rectal cancer, especially in patients with risk factors for LPLN metastasis (10–12). On the contrary, in Western countries, neoadjuvant treatment followed by TME without LPLND, which is associated with additional morbidities, is accepted as the standard of care for locally advanced rectal cancer (6, 7). Some researchers recommend selective LPLND when performing TME after neoadjuvant treatment in rectal cancer patients at high risk for LPLN metastasis (13, 14).

Herein, we review the latest relevant studies regarding the optimal management of LPLN, suggest the most appropriate policies according to current evidence, and discuss future research directions.

## Anatomy and lymphatic drainage of the rectum

**Figure 1:**
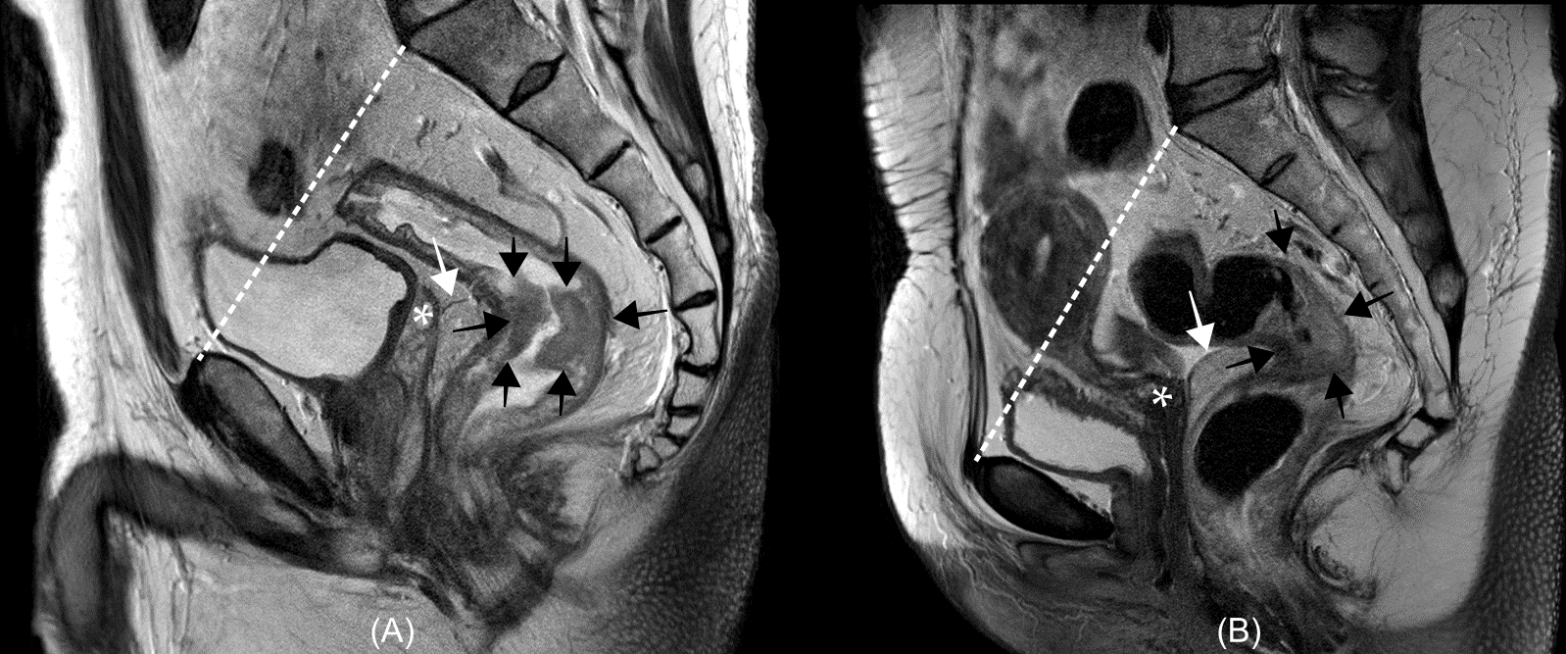
Examples of rectum anatomy on magnetic resonance image from a man **(A)** and a woman **(B)** with rectal cancer. In the National Comprehensive Cancer Network (NCCN) guidelines, the rectum is defined as the portion of the bowel located below an imaginary line drawn from the sacral promontory to the top of the pubic symphysis (white dashed lines). The anterior peritoneal reflections (white arrows) is located around the upper border of the seminal vesicle [asterisk in **(A)**] in the man or uterocervical angle [asterisk in **(B)**] in the woman. In the NCCN guidelines, the rectum is divided according to the relative location from the anterior peritoneal reflection: upper rectum, above the anterior peritoneal reflection; mid-rectum, at the anterior peritoneal reflection; and lower-rectum, below the anterior peritoneal reflection. Rectal cancers (black arrows) locate below **(A)** and across **(B)** the anterior peritoneal reflection.

The rectum is the last portion of the large intestine, which connects the sigmoid colon to the anal canal. The length of the rectum is approximately 12–15 cm, measured from the anorectal ring to the rectosigmoid junction, which is approximately at the same level as the sacral promontory (15, 16). Although there are several classifications of the rectum anatomy, the rectum has been traditionally divided into three parts: upper, middle, and lower according to the length or relative locations from the peritoneum which covers the upper third of the rectum anteriorly and laterally, and the mid-third rectum only anteriorly, and does not cover the lower third (15). Although there are no strictly established criteria for the subdivision of the rectum, the recent National Comprehensive Cancer Network guideline emphasized the use of magnetic resonance imaging (MRI) in the definition of the rectum which is the portion of the bowel located below the pelvic inlet (an imaginary line drawn from the sacral promontory to the top of the pubic symphysis) and divides the rectum as follows: upper rectum, above the anterior peritoneal reflection; mid-rectum, at the anterior peritoneal reflection; and lower-rectum, below the anterior peritoneal reflection (**Figure 1**) (17).

**Figure 2:**
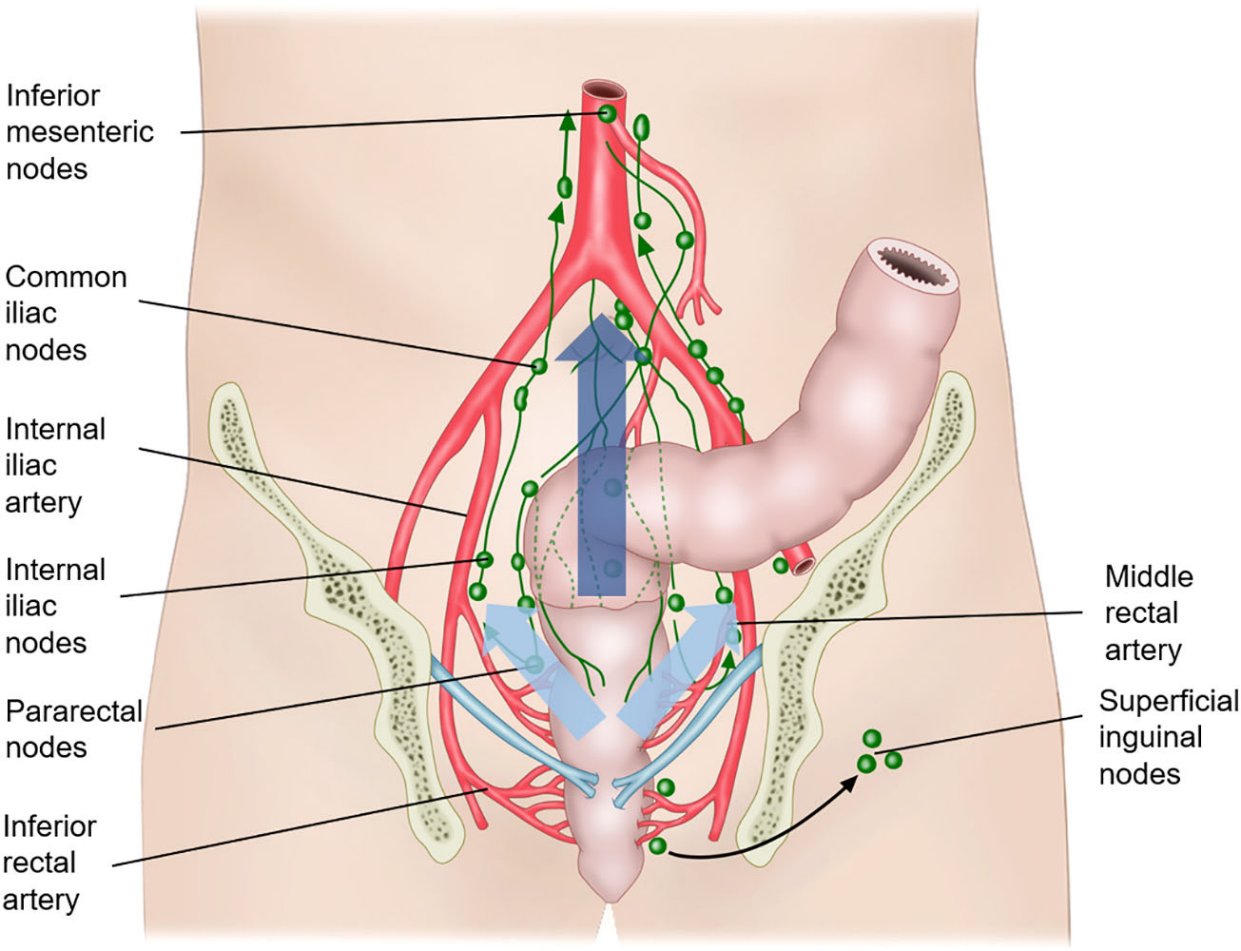
Lymphatic drainage of the rectum. The lymphatic drainage above the peritoneal reflection follows mostly an upward pathway along the perirectal, superior rectal, and inferior mesenteric nodes *via* the mesenteric vessel (along the dark blue arrow). Rectal tumors below the peritoneal reflection tend to drain along the mid-rectal vessel and then into the pelvic sidewall lymph nodes, including the obturator, internal iliac, external iliac, and common iliac lymph nodes (along the sky-blue arrow). The third route of the lymphatic drainage is from the level below the dentate line, in which the lymphatic spread is along the inferior rectal vessel and then into the superficial inguinal and external iliac lymph nodes.

Lymphatic drainage of the rectum depends on the rectal level. **Figure 2** shows the lymphatic drainage of the rectum according to the location. Lymphatic drainage above the peritoneal reflection follows mostly an upward pathway along the perirectal, superior rectal, and inferior mesenteric nodes *via* the mesenteric vessel (15). The pelvic sidewall nodal drainage of rectal tumors above the peritoneal reflection is very low at 1.5%–3.6% (15). Rectal tumors below the peritoneal reflection tend to drain along the mid-rectal vessel and then into the pelvic sidewall lymph nodes, including the obturator, internal iliac, external iliac, and common iliac lymph nodes (18). The third route of the lymphatic drainage is from the level below the dentate line, in which the lymphatic spread is along the inferior rectal vessel and then into the superficial inguinal and external iliac lymph nodes.

## Clinical implication of LPLN metastasis

The clinical implications of LPLN metastasis from rectal cancer regarding the risk of locoregional recurrence in rectal cancer have been discussed in various reports. Although the rates of locoregional recurrence in rectal cancer are not relatively high (approximately less than 10% after neoadjuvant chemoradiation [NCRT] followed by TME), more than half of locoregional recurrences occur in the lateral pelvic sidewall. Kim et al. analyzed the location of locoregional recurrences in rectal cancer after NCRT and TME in 412 patients (19). The researchers reported that the rate of locoregional recurrence was 7.9%, in which the lateral pelvic recurrence consisted of 82.7% of total locoregional recurrence. In this study, the existence and size of clinically suspicious LPLN metastasis were significantly associated with locoregional recurrence of up to 50%–87.5% for LPLNs with a size of 10 mm or more. LPLN metastasis is a prognostic factor not only for locoregional recurrence but also for overall survival. The 5-year overall survival rate was as low as 25%, which is much lower than that of patients without LPLN metastasis.

The reported prevalence of LPLN metastasis in locally advanced rectal cancer ranges from 7% to 50% (9). The wide range in the prevalence of LPLN metastasis reflects the heterogeneity in the study populations among studies. However, particular populations have the potential for LPLN metastasis, and considering their poor prognosis, comprehensive strategies, including the selection of high-risk populations and management additional to NCRT and TME (which is the current standard for locally advanced rectal cancer), need to be established.

## Risk factors and clinical detection of LPLN metastasis

Because of the differences in lymphatic drainage according to the level of the rectum, the position of the rectal cancer is a well-known risk factor for LPLN metastasis, with the lower positions posing the highest risk. In a previous study, for locally advanced rectal cancer at T3 or T4, LPLN metastases were found in 20.1% of patients with low rectal cancer, but only in 8.8% of those with upper rectal cancer (20). Besides the lower location of rectal cancer, various factors including the female sex, moderate to poor differentiation, advanced T stage, tumor size of 4 cm or more, lymphatic invasion, and clinically positive LPLN have been identified as risk factors for LPLN metastasis (8). In particular, low rectal cancer with multiple risk factors show a high rate of LPLN metastasis of up to 49% (20).

As a clinically positive LPLN is a risk factor, the identification of metastatic LPLN on diagnostic images plays an important role. Various imaging modalities, including ultrasonography, pelvic computed tomography (CT), positron emission tomography (PET)-CT, and MRI can be applied to evaluate pelvic lymph nodes (21–23). CT and MRI are widely used for radiological nodal staging. There are problems in distinguishing between normal and metastatic LPLNs as LPLNs can be detected in healthy populations with sizes ranging from 7 to 10 mm in the short axis according to location (24) and consensus regarding the range for the size of LPLN metastasis is lacking (23). Ishibe et al. evaluated the diagnostic accuracy of MRI for LPLN metastasis and reported that the sensitivity, specificity, positive predictive value, negative predictive value, and accuracy of MRI were 43.8%, 98.5%, 87.5%, 88.1%, and 88.1%, respectively, with a cut-off value of 10 mm along the nodal short axis (25). Ogawa et al. used a cut-off value of 5 mm along the short axis for the criterion of LPLN metastasis on MRI and reported a diagnostic accuracy of 78.4%–80.4% (26). However, these studies showed improved performance in the prediction of LPLN metastasis by combining clinical parameters including histopathological grade and metastasis in perirectal lymph nodes (27). While these studies dealt with the radiology of LPLNs for which NCRT was not performed, the performance of MRI after NCRT in the prediction of LPLN metastasis was also evaluated. Oh et al. identified a post-NCRT LPLN size with a cut-off of 5 mm as an independent factor for LPLN metastasis, whereas pre-NCRT LNPN size was not significant in the multivariate analysis (28).

While LPLN size is mostly used for the clinical detection of LPLN metastasis, the diagnostic values of other parameters such as morphologic predictors, signal intensity on diffusion-weighted imaging, and metabolic uptake value of PET-CT in the prediction have also been evaluated in various studies (21, 23, 29). In addition, both MRI and PET-CT can be used as complementary tools for the preoperative assessment of the recurrence risk of LPLN after NCRT for rectal cancer (30). However, radiological identification of LPLN metastasis remains challenging. In addition to the radiological finding, methodologies for perioperative detection of metastatic LPLN using fluorescence imaging during surgery have been evaluated. Yasui et al. tested the feasibility of indocyanine green (ICG) fluorescence imaging in the identification of the lateral sentinel lymph node during laparoscopic LPLND for advanced lower rectal cancer (31). Similarly, Yeung et al. evaluated the performance of one-step nucleic acid assessment combined with intraoperative ICG fluorescence lymphatic mapping (32). However, these investigations are just pilot studies and much more evidence is required for the general use of perioperative fluorescence image technique.

## Management of LPLN metastasis: Surgical approach

In the 1950s, the first descriptions of LPLND were reported, and it was performed systematically in Western countries (33). However, the frequent and serious complications such as major genitourinary dysfunction, bleeding, defecatory dysfunction, longer operative time, and prolonged hospital stay were noted, and the survival benefit of LPLND was deemed insignificant (34–36). The functional outcomes tend to be improved over time. According to a systematic review from Cribb et al., the rates of urine and male sexual dysfunction are significantly lower in the contemporary cohort (patients who underwent LPLND after the year 2000) compared with past cohort (those who underwent LPLND before the year 2000) (37). This result may imply that modern surgical techniques that employ autonomic nerve preservation and unilateral dissection in LPLND potentially can reduce the functional complication. However, LPLND was frequently abandoned in Western countries. Conversely, in Japan, the importance of LPLND in rectal cancer has remained a priority, and trials of surgical approaches to control LPLN metastasis have been conducted.

Various studies comparing the TME with LPLND and TME only for rectal cancer have been conducted, mainly by Japanese groups. Those studies are summarized in **Table 1**. A meta-analysis for some of these studies, which were published before 2009, showed increased urinary and sexual dysfunction but no benefits for cancer-specific cases, including local and distant recurrence in the LPLND group. However, subsequent literature, mainly from Japan, asserted the benefit in terms of oncological outcomes of LPLND for rectal cancer (35). Ozawa et al. performed a retrospective study with a propensity score matching analysis of pT3/T4 low rectal cancer patients from a cohort of the Japanese Society for Cancer of the Colon and Rectum (JSCCR) registry (43). The authors showed a statistically significant (p = 0.013) higher overall survival rate at 5 years in the LPLND group (68.9%) than in the TME-only group (62.0%). In the subgroup analysis, the survival benefit of LPLND was significant only in the LPLN-negative group (p = 0.006) but not in the LPLN-positive group (p = 0.415). This indicates that overall survival was impacted by LPLND only in LPLN-negative pT3-4 low rectal cancer patients. Subsequently, the results of a randomized trial of TME with or without LPLND in rectal cancer were reported by the Japan Clinical Oncology Group (JCOG) (38, 45). In this study, the JCOG0212 trial examined the non-inferiority of TME alone compared to TME plus LPLND, with the primary endpoints of relapse-free survival, in patients with stage II-III rectal cancer extending below the peritoneal reflection without LPLN with a short-axis diameter of 10 mm or more on primary CT or MRI. The researchers failed to prove the statistical non-inferiority of TME alone. The rate of local recurrence was significantly lower in the TME plus LPLND group (7.4% vs. 12.6%); however, there was no significant difference in the relapse-free survival and overall survival curves between the groups. In the long-term follow-up results of JCOG0212, the non-inferiority of TME alone was not supported. In the subgroup analysis, TME plus LPLND improved relapse-free survival in the clinical stage III disease subgroup compared with TME alone. Based on these results, the JSCCR guidelines recommend LPLND even if LPLN with a short-axis diameter of 10 mm or more is not detected radiologically (12). The JCOG0212 study was the only randomized trial comparing between TME with LPLND and TME alone without neoadjuvant treatment. Although the target population of the JCOG0212 trial was the patients with LPLN-negative low rectal cancer, the trial allowed the enrollment of patients with LPLN <10 mm in short axis which could also be clinically suspicious LPLN metastasis of which the risk was at least 7.4% according to the trial, and the LPLND along with TME could be effective in the patients with suspicious LPLN of size less than 10 mm. However, the results of the trial are not applicable to rectal cancer patients with LPLN ≥10 mm in short axis on primary radiology and the role of LPLND in this population needs to be evaluated by a randomized controlled trial.

**Table 1:** Summary of the selected literatures comparing the total mesorectal excision with or without lateral pelvic lymph node dissection.

Study^*^	Design	Inclusion criteria	Intervention (n)	pLPLN (+)	Oncological outcome
Fujita et al. (38)	RCT	stage I-III, lower rectal cancer, No cLPLN metastasis	TME+LPLND (351) vs. TME (350)	7.4%	7-year RFS, 71.7% (TME+ LPLND) vs. 70.7% (TME)
Komori et al. (39)	R^†^	stage I-III, lower rectal cancer, No cLPLN metastasis	TME+LPLND (351)	7.3%	–
Kobayashi et al. (40)	R	stage I-III, lower rectal cancer	TME+LPLND (784) vs. TME (488)	14.9%	Crude LRR, 10.5% (TME+LPLND) vs. 7.4% (TME)
Akiyoshi et al. (41)	R	stage I-III, lower rectal cancer	TME+LPLND (5789) vs. TME (5778)	11.3%	3-year LRFS, 97.3% (TME+LPLND) vs. 92.9% (TME)
Kuster et al. (42)	R	stage I-III	TME+LPLND^‡^ (59) vs. TME (141)	1.9%	Crude LRR, 10.7% (TME+LPLND) vs. 0.8% (TME)
Ozawa et al. (43)	R	lower rectal cancer, pathologic T3/4	TME+LPLND (1264) vs. TME (576)	11.6%	5-year OS, 68.9% (TME+LPLND) vs. 62.0% (TME)
Oki et al. (44)	R^†^	mid-to lower rectal cancer, stage II-III	TME+LPLND (215) vs. TME (230)	–	5-year LRR, 18.5% (TME+LPLND) vs. 19.2% (TME)

*These studies enrolled the patients who did not undergo neoadjuvant chemoradiation.

†These studies are ad-hoc retrospective studies from randomized control trial.

‡The lateral pelvic lymph node dissection was performed when stage II or III disease was suspected.

n, number; pLPLN (+), lateral pelvic lymph node metastasis confirmed in the pathological evaluation; cLPLN, clinically suspected lateral pelvic lymph node; RCT, randomized controlled trial; TME, total mesorectal excision; LPLND, lateral pelvic lymph node dissection; RFS, recurrence-free survival; R, retrospective study; LRR, locoregional recurrence; LRFS, local recurrence-free survival; OS, overall survival.

The data described above did not include the use of NCRT. The role of LPLND in rectal cancer patients who underwent preoperative RT has also been also investigated in various studies which are summarized in **Table 2**. Initially, Nagawa et al. conducted a randomized controlled trial comparing the outcomes between TME with LPLND and TME only with preoperative RT (46). This study showed no differences in overall survival, disease-free survival, and recurrence rate. However, significantly higher rates of urinary and sexual function after the surgery were observed in the TME with LPLND group. This study suggested that LPLND was not necessary for patients with low rectal cancer who underwent preoperative RT. Conversely, a study by Ogura et al. showed the necessity of LPLND in addition to NCRT for the reduction of the lateral local recurrence rate (13). This study reported that while LPLND did not reduce the lateral local recurrence, local recurrence, distant recurrence, or cancer-specific survival in the total cohort, in the subgroup with LPLN ≥7 mm in short axis on primary MRI, NCRT plus TME with LPLND resulted in a significantly lower 5-year lateral local recurrence of 5.7% compared with NCRT plus TME alone (19.5%, p = 0.042). In addition, a more recent study by Schaap et al. emphasized the size of LPLN after neoadjuvant (chemo)radiation (N(C)RT) as a predictor of lateral local recurrence (53). LPLND improved the local control in persistent internal iliac nodes after N(C)RT. In this study, LPLND also did not reduce the lateral local recurrence in the total cohort; however, local control was improved by LPLND in patients with persistent internal iliac nodes after N(C)RT. Similarly, Malakorn et al. evaluated the pathologic positivity of LPLN after NCRT and TME plus LPLND for rectal cancer patients. In this study, there was no metastatic LPLNs in surgical specimens for the LPLN with short axis less than 5 mm on post-NCRT MRI and no patients with positive LPLNs developed lateral compartment recurrence (55). Ogura et al. also reported that the size reduction of LPLN from a short-axis of 7 mm or greater on primary MRI to that of 4 mm or less on post-NCRT MRI abolished the risk of LPLN recurrence, and LPLND can be avoided. However, in persistently enlarged LPLNs greater than 4 mm in the internal iliac compartment on post-NCRT MRI, the risk of LPLN recurrence was high, and an LLND lowered this risk significantly (56).

**Table 2:** Summary of the selected literatures comparing the total mesorectal excision with or without lateral pelvic lymph node dissection after neoadjuvant (chemo) radiation.

Study	Design	Inclusion criteria	Intervention (n)	pLPLN (+)	Oncological outcome
Nagawa et al. (46)	RCT	resectable lower rectal cancer	TME+LPLND (23) vs. TME (22)		5-year OS, 68.9% (TME+LPLND) vs. 62.0% (TME)
Akiyoshi et al. (47)	R	stage II-III, lower rectal cancer	TME+LPLND (38) vs. TME (89)	65.8%	3-year LPLN RFS 97.3% (TME+LPLND) vs. 92.9% (TME)
Ishihara et al. (48)	R	clinical T2-4	TME+LPLND (31) vs. TME (191)	51.6%	Crude LRR, 0.0% (TME+LPLND) vs. 1.0% (TME)
Kim et al. (49)	R	clinical T3-4, mid-to-lower rectal cancer	TME+LPLND (30) vs. TME (31)	16.7%	3-year LPLN RFS 100% (TME+LPLND) vs. 76.9% (TME)
Ogura et al. (50)	R	lower rectal cancer	TME+LPLND (107) vs. TME (220)	24.3%	3-year LPLN RFS 96.8% (TME+LPLND) vs. 94.8% (TME)
Matsuda et al. (51)	R	clinical T3-4, lower rectal cancer or N positive rectal cancer	TME+LPLND (32) vs. TME (13)	23.3%	5-year RFS 74.7% (TME+LPLND) vs. 78.6% (TME)
Konishi et al. (52)	Phase II	stage II–III lower rectal cancer and ≥ 1 of the poor-risk features on MRI^*^	TME+LPLND (30) vs. TME (13)	13.3%	3-year RFS 86% (total cohort)
Ogura et al. (13)	R	clinical T3/4	TME+LPLND (142) vs. TME (1,062)	24.6%	HR of TME alone for LPLN recurrence 0.764 (95% CI, 0.376–1.553, p = 0.457)
Schaap et al. (53)	R	clinical T3-4, lower rectal cancer	TME+LPLND (90) vs. TME (651)	51%	HR of TME+LPLN for LPLN recurrence 0.79 (95% CI, 0.36–1.73, p = 0.562)
Kawai et al. (54)	R	clinical T3-4, lower rectal cancer or N positive rectal cancer	TME+LPLND (42) vs. TME (237)	52.4%	Crude LPLN recurrence, 0.0% (TME+LPLND) vs. 1.4% (TME)

*Stages are clinical.

*cT4 disease, threatened (≤ 1 mm) or involved circumferential margin, mesorectal N2 disease, lateral nodal disease, and/or tumors requiring abdominoperineal resection due to involvement of the levator muscle or anal canal.

pLPLN (+), lateral pelvic lymph node metastasis confirmed in the pathological evaluation; RCT, randomized control trial; LPLN, lateral pelvic lymph node; TME, total mesorectal excision; LPLND, lateral pelvic lymph node dissection; OS, overall survival; R, retrospective study; RFS, recurrence-free survival; LRR, locoregional recurrence; HR, hazard ratio; CI, confidence interval.

Based on previous studies showing the heterogeneous effect of LPLND on oncological outcomes, it is difficult to assert that LPLND should be recommended uniformly for low rectal cancer with locally advanced stages. Concurrently, however, there seems to be a subgroup appropriate for LPLND even after NCRT. Therefore, efforts to establish an adequate indication for LPLND are required. Furthermore, considering the morbidity of LPLND, the application of LPLND should be decided on an individual basis by comprehensively taking into account both surgical risk and oncological benefits.

## Management of LPLN metastasis: Radiation therapy

In contrast to Japanese surgeons prioritizing the surgical clearance of LPLN, in North America and Western countries NCRT is preferred and LPLND is rarely employed unless suspected LPLN metastasis with enlarged size is persistent on imaging following NCRT (7). During preoperative irradiation, the whole pelvis, including the regional lymphatic area, is encompassed in the treatment field (46). Therefore, theoretically, there is a chance of eradicating malignant cells in the metastatic LPLN by irradiation. This hypothesis is supported by Kusters et al., who reported that preoperative RT plus TME resulted in 5-year rates of local recurrence (5.8%) and lateral pelvic recurrence (0.8%) that were comparable with those of TME with LPLND (6.9% and 2.2%, respectively) (42). Similarly, Kim et al. compared the oncological outcomes between TME with adjuvant chemoradiation and those with LPLND in stage II and III rectal cancer patients (57). The authors reported comparable survival rates in both groups, but a significantly higher locoregional recurrence rate in TME with LPLND groups. These results seem to imply that RT is sufficiently effective for LPLN management, potentially leading to the omission of LPLND.

Based on the previous results that NCRT for locally advanced rectal cancer achieved downstage of approximately 9%–31% of and 13%-20% of pathological complete response, similar rates of pathological response in LPLN are hypothetically expected (6, 58, 59). However, studies evaluating the response of LPLN after NCRT are very limited. Akiyoshi et al. observed a reduction in LN diameter of 60% in 16 out of 77 patients after NCRT with 45-50.4 Gy. The rate of metastasis in LPLN was higher in the non-responsive LPLN group (75% vs. 20%) (60). Oh et al. reported a response of LPLN to NCRT (with 50.4 Gy for the 92.4% of patients) in 30 out of 66 patients (28). The rates of metastasis in LPLN were 47.2% and 20% in persistent nodes and responsive nodes, respectively. These studies imply that the response after NCRT can be used as a predictor of LPLN metastasis, and achieving a response in LPLN after NCRT potentially leads to a reduction in the rate of LPLN metastasis.

**Figure 3:**
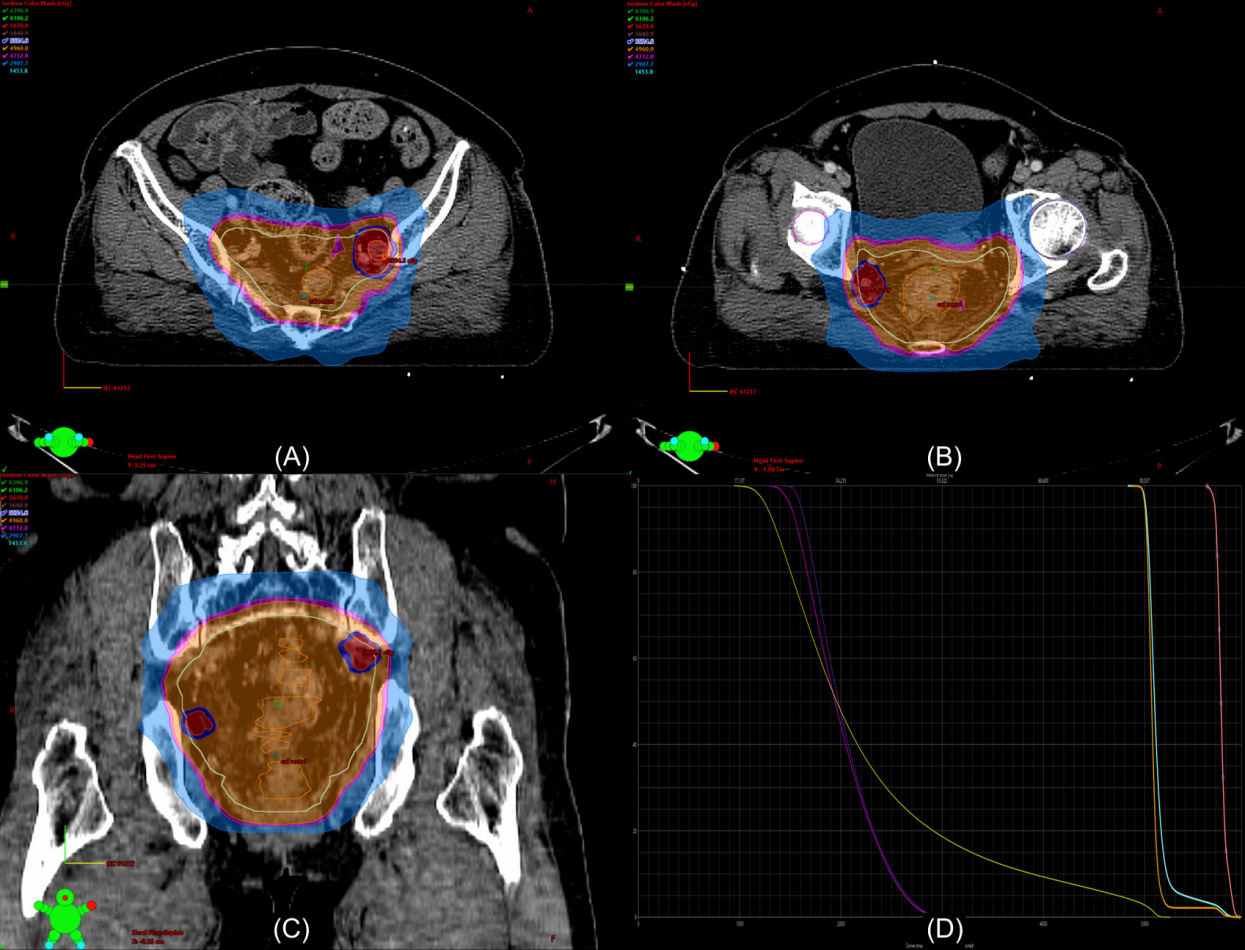
Example of a dosimetry in the radiation therapy (RT) plan with a boost to the lateral pelvic lymph node (LPLN) using the intensity-modulated RT (IMRT) simultaneous intensity boost (SIB) technique. The patient is a 62-year-old woman with non-metastatic locally advanced rectal adenocarcinoma extending to the lower rectum with perirectal fat invasion and clinically suspicious LPLN metastases in the left internal iliac and right obturator area. The LPLNs are irradiated with 58.8 Gy in 28 fractions [red color washes in **(A-C)**] while the primary tumor [orange lines in **(A-C)**] and other lymphatic area [sky-blue lines in **(A-C)**] were irradiated with 50.4 Gy in 28 fractions. **(D)** shows the dose-volume histogram of the RT plan (yellow line, bladder; purple line, right femur head; violet line, left femur head; orange line, primary tumor, sky-blue line, pelvic lymphatics; pink line, LPLNs).

Despite the efficacy of NCRT for lateral pelvic control, lateral pelvic recurrence is not a negligible problem, especially in patients with suspected large LPLN. Therefore, the intensification of treatment for lateral pelvic control seems to be required. An alternative option for avoiding LPLND after NCRT, which could lead to severe postoperative morbidities, is intensifying the RT to the suspicious LPLN metastasis by dose escalation. Clinical studies regarding dose escalation for suspicious metastatic LPLN are limited. Hartvigson et al. conducted a small-sized retrospective study to evaluate tolerance and early oncological outcomes (61). The authors performed NCRT with an additional boost to suspicious LPLN up to a range of 53.48–60.2 Gy without LPLND. Local control at 12 months was 90%, and the treatment was well-tolerated with a low risk of acute toxicity and perioperative complications. Chen et al. compared the oncological outcomes between non-boost and boost groups (62). The non-boost group contained patients without clinically involved LPLN who received NCRT with 50.4 Gy in 28 fractions. In contrast, patients in the boost group had a clinically positive LPLN and received NCRT with 50.4 Gy in 28 fractions and additional boosts to the involved LPLN up to a total dose of 54.0–59.4 Gy without LPLND. As a result, there was no significant difference in overall survival and progression-free survival between the groups. While acute grade 2 toxicity was significantly more frequent in the boost group, the rates of acute grade 3 toxicity and grade 2 and 3 chronic toxicities were comparable between the groups. The authors concluded that with an additional radiation boost, promising outcomes were observed in the boost group, which had a higher risk of LPLN metastasis and poor prognosis. Although relevant studies have been performed in very limited numbers, RT boost to suspicious LPLN metastasis can be considered as an alternative modality to LPLND. Especially, with the intensity-modulated radiation therapy (IMRT) technique, the simultaneous integrated boost (SIB) technique, which allows delivery of different doses to different target areas, leads to a dose escalation of LPLN without a significant difference in the treatment period and risk of RT-related toxicities. **Figure 3** illustrated the delivery of higher RT dose to LPLN using the SIB technique in rectal cancer with clinically suspicious LPLN.

Recently, Li et al. reported the outcome of NCRT using the SIB IMRT technique for distal rectal cancer patients who exhibited LPLN with ≥7 mm with irregular borders or mixed signal intensity on MRI. The LPLNs were irradiated with 56–60 Gy with 2.54–2.72 Gy per daily fraction while the gross primary tumor and other pelvic areas were simultaneously irradiated with 50.6 Gy and 41.8 Gy, respectively. The researchers reported the 2-year progression-free survival (PFS) rates as 85.6% even without further operative management patients, and for patients who achieved complete clinical response, the two-year PFS was 90.0% (63). The relevant data is still quite limiting; therefore, the accumulation of experiences regarding the safety of dose escalation as well as positive oncological outcomes are necessary because pelvic RT with dose escalation can potentially increase the risk of sexual dysfunction (64, 65). Comparative prospective studies between LPLND and the RT boost to the suspicious LPLN are warranted.

## Future perspectives

Although various studies have been performed and are ongoing, optimal strategies for the management of LPLN metastasis remain contradictory. In particular, the benefit of additional LPLND to NCRT plus TME is unclear. To clarify this issue, a randomized controlled trial is ongoing (66). The study enrolled advanced rectal cancer patients with suspicious LPLN metastasis who were eligible for TME and compared TME alone or TME with LPLND after NCRT. It is expected that the results will provide important information regarding the clinical usefulness of additional LPLND. However, the clinical trial is adopting a dose regimen of NCRT with 50.4 Gy in 28 fractions and does not include RT boosting. Considering the applicable dose escalation strategy for NCRT, the role of LPLND will still need further evaluation even after the publication of the clinical trial results.

With the introduction of total neoadjuvant therapy (TNT), the trend of neoadjuvant therapy for locally advanced rectal cancer patients has been changed. The TNT strategy applies a standard-dose polychemotherapy before or after NCRT in these patients and shows not only an improved disease-free survival but also higher rates of pathological complete response compared with conventional NCRT (67, 68). In a study conducted by Akiyoshi et al., TNT with induction systemic chemotherapy followed by NCRT showed a significantly lower risk of LPLN metastasis compared with conventional NCRT (odds ratio, 9.235, 95% confidence interval of 1.241–106.947, p = 0.0285) (60). This study may imply the potential of TNT in the reduction of risk for LPLN metastasis or recurrence. Furthermore, among the RT regimens adopted in the TNT approach, a short course of RT alone with five fractions is also available; however, there is no established dose escalation strategies in the LPLN management (67). Therefore, in the TNT era, the strategies for the management of LPLN also can be affected and the appropriate protocol of RT for LPLN management is necessary to be reestablished.

Additionally, it is necessary to consider that the decision to employ LPLND or not with TME after NCRT or whether to choose LPLND instead of NCRT, or vice versa, does not need to be mutually exclusive. Because there are yin and yang in both NCRT and LPLND, these strategies should be considered complementary to each other. Therefore, an appropriate indication should be established for the optimal choice of treatments. For example, to select patients at risk of LPLN metastasis after NCRT and TME, tools for detection or prediction of persistent LPLN metastasis are required. While the size of LPLNs on imaging is currently considered as a predictor for persistent LPLN metastasis, the prediction performance is not satisfactory (54). To improve the performance, further exploration of factors including additional image parameters from functional images or radiomics is required. A multimodal imaging approach using MRI, CT, and PET is also a worthwhile methodology to be investigated (21, 29). Additionally, comprehensive prediction models using a combination of other clinical factors, such as histological grade and tumor markers, which are associated with pelvic recurrence, and various radiological parameters are also worthy of investigation (27, 59). Prediction of pathological response is also potentially helpful in deciding whether to apply LPLND or not. If the pathological response after NCRT can be predicted before or after NCRT, the clearance of LPLN metastasis by NCRT can also be predicted, and we can select more appropriate candidates for LPLND in whom NCRT is not effective. Various radiological parameters or clinical factors, including tumor markers, immunologic status, and genetic markers are under investigation (59, 69, 70).

## Conclusions

Lateral pelvic recurrence is an important issue in the treatment of locally advanced rectal cancer patients with clinically suspicious LPLN. The optimal strategy for LPLN management remains unestablished. Although the surgical approach for clinically suspicious LPLN metastasis can be an option of LPLN management, RT is also a potentially effective one. Especially, with introduction of modern RT techniques including IMRT and SIB, safe dose escalation to the suspicious LPLN is available and improvement of LPLN control would be expected. However, the change in the trend of neoadjuvant therapy for locally advanced cancer by the introduction of TNT approaches can affect the effectiveness of current modalities for LPLN management. Therefore, relevant studies regarding the role of NCRT in the NPLN management in the TNT era are also required. Finally, NCRT and LPLND are not mutually exclusive and can be complementary tools. Because each modality has its own weakness and strength, optimal selection criteria are necessary to be established based on the risk of LPLN progression after NCRT or LPLND.

## Author contributions

Conceptualization, HP and JY; methodology, GY and JY; writing – original draft preparation, GY; writing – review and editing, HP and JY. All authors have read and agreed to the final version of the manuscript.
